# Mental health readiness as health security infrastructure: lessons from four African countries

**DOI:** 10.11604/pamj.2026.53.95.50407

**Published:** 2026-02-23

**Authors:** Eric Nzirakaindi Ikoona, Lucy Namulemo, Ronald Kaluya, Rebecca Ikoona, Foday Sahr

**Affiliations:** 1 National Public Health Agency, Freetown, Sierra Leone, United States of America; 2 Foothills Community-Based Interventions, Monticello, Kentucky, United States of America,; 3 Lindsey Wilson College, Columbia, Kentucky, United States of America,; 4 Uganda Counselling and Support Services, Kampala, Uganda,; 5 Makerere University-Johns Hopkins Research Collaboration, Kampala, Uganda

**Keywords:** Mental health readiness, emergency preparedness, health security, psychosocial support, Africa

## Abstract

Mental health and psychosocial support remain excluded from many emergency preparedness frameworks despite predictable psychological consequences of crises. This commentary presents four policy examples from African health systems arguing that mental health readiness should function as core health security infrastructure. Sierra Leone embeds mental health professionals in emergency governance; Liberia designates psychotropic medicines as essential with buffer stocks; Uganda deploys community health workers trained in mhGAP-HIG with supervision during floods; Ethiopia integrates mental health screening into chronic disease pathways. We outline a four-part implementation framework: year-round preparedness, activation within 72 hours, structured supervision during weeks one through eight, and equity safeguards. Mental health readiness stands as essential infrastructure comparable to vaccines and surveillance systems.

## Commentary

Public health emergencies are now relentless and diverse. Pandemics surge across borders-COVID-19 resulted in over 775 million confirmed cases globally [1]. Climate disasters intensify-floods, cyclones, and droughts triggered 32.6 million internal displacements in 2022, the highest figure ever recorded [2]. Armed conflicts persist. Sudan's civil war displaced over 10 million people in 2024. Disease outbreaks recur: Ebola, cholera, mpox. Each emergency, regardless of cause, produces predictable psychological consequences. Meta-analyses demonstrate that depression, anxiety, and post-traumatic stress disorder affect over 20 percent of crisis-affected populations [3]. Yet in most preparedness frameworks, infection control and logistics surge within hours while mental health and psychosocial support (MHPSS) is delayed, underfunded, or omitted entirely. This pattern is not inevitable; it represents a systems failure that can be reversed. African health systems are demonstrating that mental health readiness can be operationalised-treated not as a post-crisis add-on but as a core public health function built into emergency preparedness itself. The implications reach beyond Africa, and what these countries have learned offers a blueprint for how any nation can shift from reactivity to resilience. We use four country examples to propose a practical four-part framework for mental health readiness in emergencies.

### Four examples of readiness by design

#### Sierra Leone: mental health inside emergency command

After Ebola, Sierra Leone embedded mental health nurses into government hospitals and integrated them into the national emergency operations centre [4]. When mudslides struck Freetown in 2017, these nurses provided psychosocial support immediately, faster than external responders could mobilise. Based on our direct experience with the National Public Health Agency during this response, mental health staff were activated within hours because they were already inside the system. This integration works because incentives shift: MHPSS leaders at the operations table influence budgets, supply chains, and coordination. Routine inclusion in governance structures and simulation drills has normalised MHPSS as a core emergency function.

#### Liberia: medications as essential supplies

Following Ebola, Liberia classified fluoxetine, amitriptyline, and diazepam as essential medicines [5]. The post-Ebola investment plan established psychotropic supply chains integrated with existing pharmaceutical logistics. Based on programmatic reports, a 90-day buffer stock is now maintained and tracked alongside vaccines and antimalarials through national logistics management information systems. Stockouts appear on national dashboards monitored at district and national levels. No separate system or special funds are required-psychotropics became routine commodities. The result is continuity of care: providers can treat without interruption during crises.

#### Uganda: supervision unlocks effectiveness

During Uganda's 2020 floods, community health workers trained in the WHO Mental Health Gap Action Programme-Humanitarian Intervention Guide (mhGAP-HIG) deployed rapidly in affected districts. Experience from Ugandan programmes, together with trials of psychological treatments delivered by supervised community and lay health workers, shows regular supervision strengthens quality and improves depression outcomes. Community health workers who receive structured supervision can deliver group psychological interventions that reduce depressive symptoms among caregivers and people living with HIV in routine government services [6,7]. Training without follow-up loses power; readiness requires structured oversight.

#### Ethiopia: task-sharing in chronic disease pathways

In rural Ethiopia, primary care providers were trained to detect and treat mental disorders in people with epilepsy [8]. Integrating mental health screening into existing chronic-care pathways improved access, reduced dependence on psychiatrists, and enhanced system efficiency. This approach illustrates how integration into existing chronic-care platforms can extend beyond epilepsy to other conditions such as diabetes and hypertension. The lesson is clear: embedding rather than isolating MHPSS strengthens health systems.

### A four-part implementation framework

**Year-round preparedness:** this component spans three domains. Governance: appoint national mental health emergency focal points with direct reporting lines to health ministers or emergency management directors; include MHPSS in emergency simulation drills alongside logistics and epidemiology. Medications and logistics: reclassify psychotropic medications as essential medicines and maintain 90-day buffer stocks integrated into existing supply chain monitoring. Workforce and legal environment: train community health workers annually in Psychological First Aid and mhGAP-HIG protocols; decriminalise suicide and enact safe triage protections. Based on programme budget analyses from Liberian and Ugandan initiatives, baseline preparedness costs approximately US$0.15-0.30 per capita annually.

**Activation within 72 hours:** the first 72 hours determine whether mental health becomes integrated or marginalised. Activation means launching helplines and community health worker outreach within hours of crisis onset, conducting rapid orientation sessions for frontline workers, and integrating MHPSS into existing emergency operations meetings. Screen for acute distress, suicide risk, psychosis, and severe agitation. Deploy Psychological First Aid at community level and mhGAP-HIG protocols at primary care facilities [9]. Timestamp each call and referral; link follow-up to clinical teams for continuity.

**Learning cycles during weeks 1-8:** the first eight weeks are critical for protocol refinement and quality assurance. Conduct biweekly supervision for all frontline workers involving case review, protocol adherence checks, and problem-solving. Use validated instruments such as the Patient Health Questionnaire-9 to measure progress. Track adherence to protocols and troubleshoot system bottlenecks. Where geography limits in-person supervision, mobile platforms such as WhatsApp-based case conferencing can maintain quality. Based on internal programme data from Uganda, mobile supervision costs approximately US$2 per worker per month.

**Equity and access safeguards:** emergency mental health services often replicate existing inequities. Design services that are confidential, physically accessible, and trauma-informed. Disaggregate all access metrics by age, gender, displacement status, and disability. Review weekly during the first eight weeks to ensure equitable reach to women, older adults, people with disabilities, and displaced populations. Engage organisations of persons with disabilities and women's groups in service design and monitoring to move beyond passive equity tracking toward active community participation.

### Building political will

Operational frameworks require political commitment. Three strategies have proven essential. First, reframe mental health readiness as security infrastructure rather than charitable intervention. Sierra Leone's post-Ebola integration succeeded when positioned as emergency preparedness; mental health readiness gains policy traction when framed alongside vaccines and surveillance rather than as general health system strengthening. Second, demonstrate economic returns to finance ministries. Untreated mental illness costs the global economy over US$1 trillion annually, with treatment yielding benefit-to-cost ratios of 2.3 to 5.7 depending on context [10]. In Uganda, securing approval required internal modelling showing that mental health readiness represented less than 0.5 percent of health security budgets while addressing over 20 percent of crisis health burden. Third, secure legislative rather than ministerial protection. Ministerial directives remain vulnerable to political transitions; Sierra Leone embedded MHPSS positions in civil service structures through the 2017 health workforce reform, ensuring infrastructure survival across leadership changes. The specific mix of legal and financial levers will differ by country, but the principles of security framing, economic argument, and legislative protection remain generalisable.

## Conclusion

Mental health readiness is health infrastructure, as essential to health security as vaccines, surveillance systems, or water treatment. It is not a post-crisis luxury but a foundation of resilience. The African experience demonstrates that with governance integration, steady supervision, and equity safeguards, MHPSS can transition from reactive aid to preventive infrastructure. Now is the time to embed mental health into every country's preparedness plan: act early, activate fast, supervise consistently, and protect equitably.
